# Interplay of Support, Comparison, and Surveillance in Social Media Weight Management Interventions: Qualitative Study

**DOI:** 10.2196/19239

**Published:** 2021-03-01

**Authors:** Leanne Chang, Kaushik Chattopadhyay, Jialin Li, Miao Xu, Li Li

**Affiliations:** 1 Department of Communication Studies School of Communication Hong Kong Baptist University Hong Kong Hong Kong; 2 Division of Epidemiology and Public Health School of Medicine University of Nottingham Nottingham United Kingdom; 3 Department of Endocrinology and Metabolism Ningbo First Hospital Ningbo China

**Keywords:** obesity, social comparison, social media, social support, surveillance, weight control

## Abstract

**Background:**

There has been a significant increase in the trend of using social media as a platform to deliver weight management interventions. This illustrates a need to develop a holistic understanding of doctor-patient communication and peer-to-peer communication in social media interventions and to determine their influences on weight management for people with overweight or obesity. Such studies will highlight how social media can be more effectively integrated into weight management programs to enhance individuals’ short-term and long-term weight management behaviors.

**Objective:**

The aim of this study was to examine patients’ experiences with doctor-patient communication and peer interactions in a social media–based (WeChat) weight management program, and to describe the interplay of three social influence factors—social support, social comparison, and surveillance—in their weight control practices. The program, designed and implemented by the research team located in a tertiary referral hospital in a southeastern province in China, included both diet and physical activity components that targeted people with overweight or obesity.

**Methods:**

We conducted in-depth interviews with 32 program participants of different ages (mean 35.6, SD 7.7 years), gender (18 women), duration of program membership (mean 1.4 years), and weight loss outcomes (54% weight loss to 9% weight gain). All interview data were audio-recorded, transcribed, and translated using the translation-backtranslation technique. Nvivo software was used to facilitate the coding process.

**Results:**

Results of thematic analysis indicated the distinct functions of professionally led support and peer support. Professional support was presented in the form of knowledge infusion, efficacy enhancement, and provision of timely feedback. Peer support fostered empathy and sense of belonging, and had a mutually reinforcing relationship with peer comparison and peer-based surveillance. Peer comparison enhanced motivation and positive competition. However, it also reinforced negative group norms, and resulted in downturns in reference standards and collective inactivity. Social media surveillance prompted participants’ reactions to the gaze from medical professionals and peers that could be encouraging or inhibiting. Surveillance enhanced vigilance with weight control norms; however, its influence weakened when participants chose to fake weight data and turn off notifications. Findings from this study illustrated the interrelated and fluctuating influences of support, comparison, and surveillance.

**Conclusions:**

The interactive traits of social media eased the practices of social support and social comparison, and created new forms of surveillance. This study contributes to an in-depth understanding of social media influences on individuals’ weight control behaviors. Practical implications of the study concern improved strategies for maintaining the positive dynamics of social media interactions and preventing negative resistance to surveillance technology.

**Trial Registration:**

Chinese Clinical Trial Registry ChiCTR1900025861; http://www.chictr.org.cn/showprojen.aspx?proj=42497

## Introduction

### Background

In China, an estimated 32.3% of the adult population had overweight or obesity in 2016 [1]. This number has doubled since 1992, and China is now alongside the United States as the two nations with the largest percentage of citizens affected by overweight and obesity [2]. Standard obesity prevention and treatment focuses on improving individuals’ self-regulation of dietary regimens and physical activity [3,4]. In recent years, there has been an increasing trend of integrating social media into weight management interventions to improve participant engagement and retention [5,6]. These social media platforms include blogs, discussion forums, social networking sites, and other online communities using Web 2.0 technologies permissive for user-generated content and information exchange [7,8].

Social media has demonstrated the potential to facilitate weight management through improving provider-patient communication and peer-to-peer communication in cyberspace [8,9]. A review of the literature indicated that integrating social media into weight management interventions may increase participants’ direct interactions with health professionals and deepen provider-patient relationships [9]. Social media may enhance connections among users with common interests in weight control and amount to a new phenomenon of “peer-to-peer health care” [8]. A scoping review of social media in dietetic practices found that being able to ask for help from health professionals and perceived support from peers were important facilitators to participant engagement and dietary behaviors [6]. However, two systematic reviews of randomized controlled trials reached the same conclusion of no direct impact of social media use on weight outcomes [10,11]. One possible explanation is that social media may promote initial changes but cannot sustain them in the long run [11]. Hence, finer details are needed to understand the complex long-term influences of social media interactions [10].

Noting the complexities of social media dynamics, this study adopted a holistic approach to examining the influences of social media interactions on weight management. Specifically, this study examined three types of social influences—social support, social comparison, and surveillance—that may arise from two types of social media interactions: doctor-patient communication and peer interactions. By examining participants of a social media–based weight management program that has lasted for more than 2 years, this study contributes to addressing many of the challenges with interventions over short time frames [11]. The focus on a Chinese social media app used among a Chinese population where obesity is on the rise also adds new knowledge to the current body of literature on social media weight management interventions.

### Social Support

Social support refers to resources available from one’s social network that are intended to be helpful [12]. The support resources could be informational (eg, advice and knowledge), instrumental (eg, material and financial aid), emotional (eg, encouragement and empathy), and appraisal (eg, affirmation and evaluative feedback) [13]. Conceptual studies suggest that support from health professionals and peers differs in their functions and ways of promoting behavioral change [14,15]. Professionally led support may facilitate health behaviors through skills training, monitoring, and feedback that appear to be directive, prescriptive, and guided by rules [14]. Peer support may promote self-regulation through social norms, identity, and companionship, and appear to be noncompulsory, mutual, and decentralized [14].

Social support is not a new concept in health interventions. However, limited studies have examined both professional support and peer support in social media weight management interventions. One field experiment compared 425 users’ submission of dietary diaries and found that professional support had positive effects on diary submission, whereas peer support curbed user participation [16]. The negative effect of peer support was attributed to the perceived threats from fellow users who reported better dietary practices [16]. Another clinical trial with 301 women controlled for the inclusion of professional support and peer support in a web-based weight management intervention. No group difference in weight change was found, although the professional support group showed longer retention than the peer support group [17]. A focus group study including 35 adults with overweight or obesity revealed that peer support provided companionship, while professional support provided tailored instructions. However, their impacts on weight control were unexplored [18]. These initial findings guide further investigation of the coexistence of the two types of social media support and their interplay in weight management.

### Social Comparison

Social comparison involves one’s comparison to similar others for maintaining a stable view of oneself [19]. The concept of online social comparison has been widely studied in the literature on social media and body image, highlighting its negative effects on weight concern and disordered eating [20]. Recently, more research has looked into the positive impacts of social comparison on weight loss [9]. An experiment on a Facebook exercise app indicated that when users were allowed to track each other’s physical activity records and progress, they exercised more than those in the nonsocial conditions. The benefits of positive competition were emphasized in user feedback [21]. In a large-scale online weight management program, the design of within-team and among-team competitions improved participants’ overall level of physical activity, although the specific mechanisms by which social comparison exerted these influences were unspecified [22]. A metareview of 26 studies of physical activity apps found that modeling, information sharing, and social networking may be the underlying factors prompting online social comparison [23]. One suggestion derived from the metareview was to investigate different dimensions of social comparison behaviors and their relationships with other social factors [23]. An interview study of user perspectives on a fitness app revealed that social comparison motivated some users to self-improve and be physically active; however, it also demotivated others when they were unable to catch up with high performers [24]. These findings pave ways for further exploration of how social comparison intervenes in online social networking and influences weight management in possible positive and negative ways.

### Surveillance

Surveillance refers to the collection and monitoring of personal details to manage a population [25]. Foucauldian-based interpretation often uses the panopticon metaphor to describe surveillance as a form of control conducted through self-awareness and self-monitoring [26,27]. The panopticon is a theoretical prison design that places the guard tower at the center and the prisoners at the surrounding cells; the prisoners’ awareness of the gaze from the invisible guards and their internalization of the norms explain their self-regulated behaviors [28]. On social media, users are both observers and observees who can track each other’s personal information and be surveilled [29]. The unilateral communication taking place on social media fosters new practices of participatory surveillance that involve “many watching many others” among users, which differs from the conventional form of surveillance involving a few people of authority watching many observees [26,30].

Limited studies have examined participatory surveillance in weight-related social media interventions. One case study described that using social media to report daily health routines such as exercise, diet, and blood glucose level encouraged users with chronic illness to persist in self-health management [31]. The presence of an audience and feedback via social media enhanced self-surveillance, and one’s continual practices and reporting of health behaviors [31]. Two focus group studies of school children’s use of fitness apps indicated that displaying a positive image under the peer gaze was a major motivator for fitness pursuits and constant self-monitoring [27,32]. Adolescent users reported pressure to conform to fitness norms and perform self-tracking with the awareness of being watched [27,32]. Critical scholars have raised concerns about the negative outcomes of surveillance, such as invasion of privacy and resistance [26,30]. However, surveillance may also evoke agency and better self-monitoring of health [29]. More empirical evidence is needed on surveillance in social media interactions.

Based on this background, in this study, we sought to explore the cybersocial influences of social media interactions on weight management behaviors among people with overweight or obesity. The research question was as follows: How do social media support, comparison, and surveillance affect weight management among people with overweight or obesity?

## Methods

### Research Context

The research context involved a free-of-charge WeChat-based weight management program implemented by a tertiary referral hospital in an urban city with a population of 8 million inhabitants in southeast China. WeChat is a multipurpose messaging, mobile payment, and social media app that functions similarly to the combination of Facebook and Apple Pay. By 2016, WeChat had reported a penetration rate of 93% in first-tier cities and an estimated penetration rate of 80% across the population in China [33,34]. Its prevalence made it an easy-to-use intervention delivery tool in the studied context.

**Figure 1:**
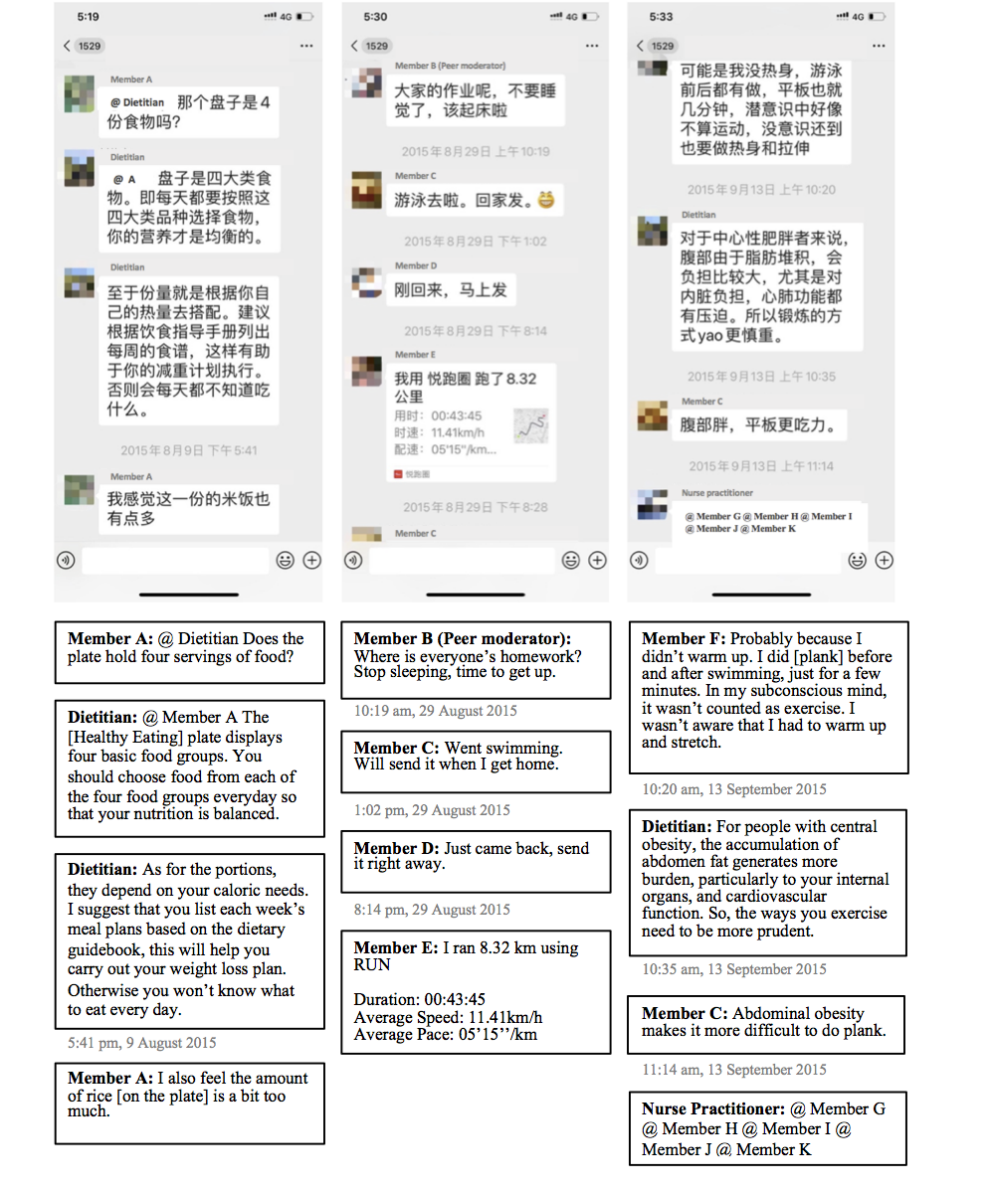
Screenshots of interactions in a private WeChat group.

Starting from July 2015, the program, which features scientific weight loss for people with overweight or obesity, was promoted through local television programs, newspapers, hospital websites, WeChat groups, and doctor recommendations during outpatient appointments. Interested individuals registered with the hospital and attended a weight management workshop to learn the basics of healthy eating (eg, nutrients and calories), exercise (eg, frequency, intensity, and duration), and program logistics in detail. Participants then enrolled in a WeChat private group with 10 to 12 members based on their joining time. An additional WeChat public group was made accessible to all participants. All participants used their extant WeChat account with their preferred display names to interact in the groups. Within each private group, a registered dietitian sent messages at agreed-upon times that asked participants to post their weight, dietary intake, and physical activity on a daily basis, and provided personalized feedback during the first month. Subsequently, participants were asked to report their weight on a weekly basis during the second and third months, and on a monthly basis from the fourth month onward. The three-phase design emphasized different goals (eg, from heightening awareness to weight loss maintenance) in different phases [35,36]. Two volunteer peer moderators assisted in sending reminders and collecting weight data from fellow members. A medical team of three registered dietitians, four endocrinologists, three orthopedists, and one nurse practitioner was introduced to the participants during the workshop and enrolled in all WeChat groups with their real names. All private and public groups remained open once established, wherein participants and medical professionals could freely interact with each other by posting content, commenting, and tagging a specific person to reply (Figure 1). At the time of the study, 28 private WeChat groups were created, with the oldest group being active for 2 years and the most recent for about 1 week.

### Procedures

All study procedures obtained ethical approval from the Research Ethics Panel at the University of Nottingham Ningbo China and the Research Ethics Committee at Ningbo First Hospital (Approval ID: 2017-R049). The interview participants were purposively selected with variations in gender, age, duration of membership, and amount of weight loss to allow for exploration of diverse experiences [37]. Recruitment was conducted through phone calls and face-to-face invitation during hospital visits in May and July 2017. All interviews were conducted in Mandarin by the first author between June and July 2017, and were scheduled at times convenient for the participants in a consultation room in the hospital. Participants were informed about the recruitment criteria, purpose of the study, interview process, nature of the questions to be asked, and their right to participate or withdraw on a voluntary basis. Each participant provided written informed consent prior to the interview and received one hand towel set and a free body composition analysis at the end of the interview as a token of appreciation for their time. All participants completed the interview with the knowledge that they could stop at any time if they preferred to do so.

The interviews were semistructured, beginning with a short demographic survey and general questions such as “What were the reasons that motivated you to join the weight management program?” and “What health changes have you observed since joining the program?” Participants were then asked to share their weight control practices, perceived benefits and barriers to adhering to health suggestions, observations of peer interactions and doctor-patient communication in WeChat groups, and experience with support exchange among group members and support sharing from the medical team. Each interview lasted about 1 hour (mean 57.3 minutes, SD 7.0, range 45-75). After 32 interviews, the research team concluded that we had reached the point of data saturation after observing replications in the data and identifying no new ideas [38]. All interviews were transcribed verbatim by the first author and three student assistants in Mandarin, and then translated by four professional translators into English using the translation-backtranslation technique.

### Participants

Thirty-two participants (14 men and 18 women) from 18 different WeChat groups completed the interviews. Their ages ranged from 21 to 53 years (mean 35.6, SD 7.7 years). Six participants served as peer volunteers and the rest were group members. The average duration of program participation was 1.4 years (mean 510.6 days, SD 203.4). The absolute weight change ranged from losing 55 kilograms to gaining 7 kilograms (mean 11.2 kilogram loss, SD 13.1). The percent weight change ranged from a weight loss of 54% to a weight gain of 9% (mean 12% loss, SD 13.3). Participants’ demographics are shown in Multimedia Appendix 1.

### Data Analysis

A thematic approach was used to identify themes [39]. The first author used Nvivo software to perform line-by-line analysis and generate initial codes. Through sorting, resorting, and iterative discussions, the first and second authors grouped interpretive categories into potential themes that addressed the research question. Finally, all authors examined the themes and agreed on their internal consistency and inclusion of explanatory accounts. Expert check, member check, and triangulation techniques were used to verify the validity of the results [40,41]. One dietitian and two participants reviewed the themes and agreed on the interpretations. The first author triangulated the results with 9 months of observations in the public WeChat group between August 2017 and April 2018 to validate the comprehensiveness of the findings.

## Results

### Overall Themes

Three themes were extracted to summarize the influences of social media interactions on participants’ weight management: professionally led support for capacity building, cooperation and resistance to surveillance, and mutual reinforcement of peer influences.

### Professionally Led Support for Capacity Building

Professionally led support was reflected in knowledge infusion, efficacy enhancement, and the availability of timely feedback. Table 1 presents the supporting quotes for this theme. Participants indicated that information from medical professionals corrected their misunderstanding about diet, exercise, metabolism, and ways to lose weight (quote 1). They shared common experiences of being exposed to unregulated drug advertisements, specious information about diet and exercise, and weight loss scams that circulated on different media or through personal networks and neighborhood pharmacies (quote 2). Without knowing the effects and side effects, they often tried these advertised drugs and weight loss packages, and learned their lessons only after feeling ill or paying money for products that did not work. Having a platform to communicate with medical professionals provided a reliable means to filter out false information and transform them “from amateur to academic.”

Information and feedback from medical professionals helped participants set realistic expectations, assure certainty, and enhance their efficacy in coping with weight fluctuations (quotes 3-4). This support built participant confidence such as “as long as I keep myself on the right track, I will eventually lose weight.” All participants shared that they had tried extreme weight loss methods such as consuming unregistered diet pills or fasting and observed no apparent effect. With support from medical professionals, they “became more rational” and could “stay calm even if I regain some weight.”

The availability of timely feedback reflected another aspect of receiving professional support. Communicating via WeChat allowed participants to seek additional advice and clarification that was overlooked during hospital visits. Some minor problems could be solved quickly through real-time processing or asynchronous communication (quote 5). Participants were able to receive feedback within a few hours rather than waiting until the next doctor’s appointment. Additionally, they could collectively learn from medical professionals or peer responses to avoid repetitive questioning (quote 6). The approachability of medical professionals also had a spillover effect of emotional support. WeChat communication shortened the relational distance between participants and doctors, thereby making the doctor-patient relationship “more like a friendship.” When the doctors became friends rather than faceless figures, the strengthened bonds offered more emotional support and incentives for adherence (quotes 7-8).

However, professionally led support also had limitations, which were reflected in participants’ use of knowledge gain as an excuse for procrastination, and the gap between knowing and doing. Once participants believed that they knew how to lose weight, the illusions may foster their decision to “take it easy” and “postpone the actual implementation of the process.” Factors such as structural constraints (eg, family dietary habits and the business drinking culture), low perceived vulnerability, and hesitancy to curb old habits also discounted the impact of professionally led support (quotes 9-11).

**Table 1:** Supporting quotes for theme 1: Professionally led support for capacity building.

Subtheme	Quotes
Knowledge infusion	Quote 1: “She [dietitian] didn’t give all the suggestions at once. Rather, she did this through day-to-day conversations, like making progress steadily in a quiet way. Some tips on a day and several on another, and in the end, we would bear in mind what is right and what is not.” Quote 2: “There are advertisements about diet pills on the internet boasting about their effectiveness, but they don’t mention the side effects. They claimed to be imported from Germany but, in fact, they were produced by small local food plants. There are many similar medicines on the internet. We can distinguish them by asking the doctors.”
Efficacy enhancement	Quote 3: “The doctors would tell you that this was a normal bottleneck, especially after the early period of rapid weight loss. There’ll be good results afterward. As everything is explained from a professional standpoint, it’s easier to believe.” Quote 4: “What I learned is a scientific and realistic approach to losing weight. Previously, we only knew that we should eat less, like dumping staple food. It worked, but things would go back quickly, and the effect just didn’t last. They helped us develop new eating habits, and the biggest change is that I now know what food does me good and what does me harm.”
Timely feedback	Quote 5: “If I forget to ask, I can ask them on WeChat and receive the answer soon. Otherwise, I have to wait until I meet the doctor again.” Quote 6: “You can see how team members handled their situations…and the doctors did not need to repeat the same content to every group member.”
Emotional support	Quote 7: “Our membership in the group is not like the doctor-patient relationship in a hospital. It is like becoming a friend to the medical team. Normally the doctor would wear a white gown, sitting there to examine you, but joining this group is different. The feeling is different.” Quote 8: “Because you trusted them and could sense their sincerity, you would be more willing to follow their suggestions.”
Inhibiting factors	Quote 9: “In China, people connect with each other often by having dinner or lunch together. It’s the eating culture here. It’s like people gather not to eat but to meet each other. I don’t want others to realize that I’m thinking negative about the food. It’s a bit tiring to keep doing so and it’s easy to go back to old habits.” Quote 10: “I have high cholesterol, blood pressure, and blood sugar, but I don’t feel any physical pain. This leads to the fact that I fail to value the importance of losing weight. I have the knowledge but I’m in my twenties and you ask me to eat bland food. It’s quite hard to do so.” Quote 11: “I think if I completely refuse to eat what I like, that’s a terrible life. Now I’m sort of thinking that a healthy mentality is much more important than a healthy lifestyle.”

### Cooperation and Resistance to Surveillance

WeChat created a surveillant environment that underpinned the perceived need to practice healthy eating and exercise behaviors. Table 2 presents the supporting quotes for this theme. The presence of medical professionals in the groups and an anticipation of their evaluations constantly reminded participants about being in the process of weight control and the need to self-regulate their behaviors (quotes 12-13).

However, since surveillance was performed in cyberspace, it lacked a mechanism to verify untruthful data. Five participants explicitly described the experience of reporting fake body weight and calorie intake to save face and avoid blame (quotes 14-16). When weight loss stalled, participants were lured into faking weight data with the hope that they could catch up to the falsely reported number at a later date. Once they failed to reach the desired figures, frustration and embarrassment could drive them to hide their weight loss outcomes, either by staying silent in the groups or repeating false information to make their weight data look acceptable. In most of these cases, insignificant weight loss and disengagement became a vicious circle of ineffective weight management.

Resistance to surveillance was also present in participants’ reactions to frequent WeChat notifications. Participants wanted to attend to group messages and gain information. A vibrant online community encouraged their engagement. However, message posting, replying, and tagging could be initiated by anyone at any time (Figure 1). This resulted in a large number of push notifications that were perceived to be irrelevant and disturbed participants’ daily activities or rest (quotes 17-18). Many participants reported the experience of neglecting all messages or resisting reporting weight data out of indignation. Some “muted all notifications” from the program or blocked the WeChat groups “to concentrate on work.” However, many forgot to unmute the notifications and gradually disengaged from their groups. Once participants left the virtual panopticon, surveillance lost its influence.

**Table 2:** Supporting quotes for theme 2: Cooperation and resistance to surveillance.

Subtheme	Quotes
Self-awareness	Quote 12: “As I had tried various ways to lose weight by myself using all kinds of methods and still couldn’t control myself, I knew it would be better with someone watching you. Human beings are like that. When you’re under supervision, you’ll be mindful of what you’re doing.” Quote 13: “I felt I had to monitor myself. Self-management mattered because others were watching.”
Untruthful data	Quote 14: “I needed to write lower numbers. Otherwise, the doctors would blame me.” Quote 15: “When I didn’t lose weight for 1 week, I felt embarrassed. I reported a figure and thought that I’d achieve it the next week, but I didn’t. Then, the entire process was repeated the following week.” Quote 16: “What we saw for a period of time was that everyone’s weight records were about the same as they had always been, with no increase or decrease, when everyone was actually relapsing to some degree.”
Invasive technology	Quote 17: “It seems that they’re reckless with the notification times. For example, at 6 or 7 AM, when it is still early in the morning, my mobile phone beside my pillow would be bombarded with WeChat messages. ‘Bong, Bong, Bong, Bong,’ that’s quite annoying.” Quote 18: “Many times, those messages were sent when I was working. My work involves frequent conversations with my managers. It is unacceptable that my phone keeps buzzing.”

### Mutual Reinforcement of Peer Influences

Peer support, peer comparison, and surveillance had mutually reinforcing relationships that motivated participant engagement and health practices. Illustrative quotes for this theme are shown in Table 3. Peer support was mostly presented in the form of emotional support. Participants encountered similar inconveniences in their daily lives. Thus, they empathized with each other’s feelings and experiences (quote 19). Joining a group with others who shared a similarity created a sense of belonging that engendered peer support (quote 20). Empathy, companionship, mutual encouragement, and collective empowerment were intangible resources shared by participants that bridged their feelings of isolation. The mantra of “solidarity is strength” also fueled their motivation to change (quote 21).

**Table 3:** Supporting quotes for theme 3: Mutual reinforcement of peer influences.

Subtheme	Quotes
Sense of belonging	Quote 19: “Since we were all fat, we could really understand what we felt deep down. Our families, friends, and colleagues all have some kind of prejudice against us because we were fat. In a clothes store, I often get neglected. We all know that feeling.” Quote 20: “At home, I am the only person who is fat. Here, there are so many flabby people like me, so I don’t feel as bad about myself. It gives me some sort of encouragement. I am really lazy, but when I see others trying hard to lose weight, I feel like I cannot allow myself to be left behind.” Quote 21: “Like playing games, we are a team, and having a team is powerful. Just think about this, between one against five and five against one, which one is more powerful? Of course, it should be five against one.”
Participatory surveillance	Quote 22: “Sharing the data demonstrates what you have done to lose weight. If you only lose a little, you will feel stressed. I would be envious of those who had made good progress when I had not, and I wondered whether I could do the same as them the next time.” Quote 23: “I’d look at the ranking. There were some who had made progress but later they relapsed. I was glad to see that I relapsed just a little bit. I was kind of hoping others to do less well than me.” Quite 24: “After all, it is what I want to do. Now I’m in this group, with others closely watching my progress, but I still think this depends on me, and it has little to do with others.” Quote 25: “It is true that if I saw the records of others I would ask myself, ‘Why did I rank last?’ Even though I thought I should exercise that day, I would forget.”
Negative group norms	Quote 26: “Everyone was striving to reach the goal. But this is not an easy course. So, there were some who wanted to have a rest, and this led others to think ‘well, it might be fine if I also have a rest.’” Quote 27: “Someone would eat a little more and the rest would follow. It was inevitable that people would mutually influence each other in the group. I am the kind of person who could easily be influenced by others. Therefore, I also became less active.”
Limited topics	Quote 28: “In the early days, I would talk about what I ate, how I exercised, what effects I saw, and how I overcame difficulties. Later, everyone knew how to tackle the problems and which environmental factors caused the failure to lose weight. It reached a point where nobody could help you with anything. For example, you’re thinking about changing jobs because work-related stress was too intense for you to do anything else. Nobody could make that decision for you.” Quote 29: “We’re not really friends and thus we are shy when greeting each other. You know when people interact, we do not always go straight to the topic. When I tried to make a joke and only I responded, I would feel a bit awkward. If I wanted to raise a question about weight management, it was supposed to be the medical team who replied to me… When not talking about obesity theories, I felt that I was not familiar with anyone and should not post anything.”

Participatory surveillance and peer comparison echoed one another in WeChat communication. While participants were the subjects of others’ gaze, they also watched others self-evaluate. Seeing the progress of others drove positive competition and vicarious learning. All participants compared themselves to other members. They either speculated on others’ effectiveness in weight loss or were secretly glad after finding that they were not the weakest in the group (quotes 22-23). Insofar as peer comparison was prevalent, participants noted that weight loss was a personal journey. They ultimately focused on their strength or lack of strength to sustain self-regulated behaviors rather than on their rankings in the group (quotes 24-25).

The interview data revealed that peer interactions were a double-edged sword. Positive forces derived from the interplay of peer comparison, peer support, and mutual supervision could be motivating. However, negative group dynamics may also occur to reshape group norms and collective practices. Participants noted that weight management was a tiring and difficult task. It was unavoidable that group members influenced each other (quotes 26-27). When group interactions moved toward a negative direction, the practice of mutual supervision and comparison reinforced the perception that “everyone does it; therefore, it is okay for me, too.” Negative group norms, in turn, weakened engagement and retention.

Negative group dynamics could also result from the virtual community’s limited capacity to enhance interpersonal bonds. Despite similar weight concerns, group members differed in age, occupation, work life, and medical symptoms. Over time, the few topics around weight management generated repetitive storytelling and information exchange that were not particularly useful in solving the root problems. The saturation of resource sharing explained many group members’ regression from being open and expressive to being quiet and withdrawn (quote 28).

Finally, participants’ preference for seeking information from medical professionals formed another barrier to developing deeper bonds among peers. When participants raised a question about weight management, they expected the medical team to reply to them. When not talking about obesity-related issues, they felt that they were not familiar with anyone and were hesitant to share their thoughts and perspectives (quote 29). The issue-specific communication style and participants’ preferences for professional information sources narrowed the scope and depth of peer-to-peer communication. Peer interactions tended to dwindle after the initial excitement. Ultimately, long-term peer influences were primarily through the provision of reference points and superficial companionship that did not require deeper interpersonal ties.

In sum, our findings revealed that the cybersocial influences of social media interactions, presented in the forms of support, comparison, and surveillance, could be both positive and negative, and may change over time. Table 4 summarizes the possible influences.

**Table 4:** Social influences of social media interactions.

Dynamics			Doctor-patient communication		Peer-to-peer communication
**Support**					
	Positive	Credible information sources Uncertainty reduction and confidence enhancement Availability of timely feedback Enhanced trust and bonds		Sense of belonging Empathy Companionship and teamwork	
	Negative	Excuse for procrastination Gap between knowing and doing Centralized communication		Obstacles related to within-group heterogeneity Limited topics for conversations Unhelpful repetition of experience sharing	
**Surveillance**					
	Positive	Explicit request for adherence Enhanced vigilance for weight loss norms Basis for support-giving		Exhibition spaces of social information Source of vicarious learning Increased vigilance with disciplinary control	
	Negative	Lack of validation mechanism Resistance to invasive technology Limited binding force		Reinforcement of negative group norms Collective inactivity	
**Comparison**					
	Positive	Not applicable		Positive competition Enhanced view of oneself Realistic reference points Drive for self-improvement	
	Negative	Not applicable		Downturns in reference standards Collective tolerance for relapse Withdrawal after repeated failure in competition	

## Discussion

### Principal Findings

WeChat facilitated the medical professionals’ provision of information and tailored feedback that strengthened program participants’ capacity to manage weight based on scientific knowledge. The panoptic gaze from medical professionals and peers reminded participants about the need to conform to weight control norms. However, the lack of validation mechanisms and participant resistance to invasive technology also limited the binding force of surveillance. Intragroup similarities derived peer support, encouraged participatory surveillance, and increased comparison, which are all deemed beneficial to engagement. However, peer influences were also jeopardized by negative group dynamics and the limited scope for peer-to-peer communication.

### Comparison With Prior Work

Resonating with extant literature on social support, this study demonstrates that professional support and peer support perform different functions, and their modes of influence vary [14]. Professionally led support consisted of informational and appraisal support that were perceived to be prescriptive, directive, and affirmative. The approachability of medical professionals yielded an additional spillover effect of emotional support. Peer support mainly reflected emotional support that was mutual, noncompulsory, and empathic. The presence of medical professionals in support groups circumvented the problems of misinformation, tortuous discussions, and malicious interactions that occurred in interventions incorporating peer-to-peer communication only [12]. Professional support also reduced doubts about the perceived usefulness of support identified in previous studies [42]. However, support groups inclusive of medical professionals may invite stronger top-down control that could circumscribe the development of a vibrant online community and risk directing communication back to the conventional one-to-many model. Our results suggest that the inclusion of both professional and peer support in social media weight management interventions may diversify the provision of aid to fulfill participants’ varying support needs. Nevertheless, a fine balance should be sought between the vertical communication of professional support and the horizontal communication of peer support to benefit from social media’s multidimensionality, and to create a support network space that is both informative and participative.

Our qualitative findings offer possible explanations for the nonsignificant long-term effects of social support and the declining supportive communication over time in web-based weight management interventions found in previous studies [10,17]. Upon joining a new social media group, the knowledge gain and team support lacking in participants’ previous experiences can enhance their efficacy and motivations through strengthened supervision and encouragement, and subsequently improve their self-regulated behaviors. However, the usefulness of professional support and peer support may reach saturation when there is no room to improve participants’ knowledge level further and expand their online support networks. In this study, we specified the positive influences of support at the intellectual, psychological, and emotional levels, and we also illuminated the nuances underpinning the progressively smaller impacts of support over time. The findings of possible support saturation points, the use of knowledge gain as an excuse to delay weight loss efforts, and the variable directions of group dynamics in the evolving process of supportive communication add new information to the current understanding of support effects found in experimental studies [43-45].

This study reaffirmed the prevalence of peer comparison in cyberspace [20,23], and identified the mutual reinforcement of peer comparison, peer support, and surveillance in shaping group norms and engagement. Our findings support the notion that social media create an exhibition space that permits users to play multiple roles as supporters, comparison targets, and surveillants during group interactions [46]. In this study, the display of dietary records, weight data, and conversations increased intimacy among group members, and promoted comparison and peer-based surveillance. A supportive environment encouraged positive competition, which induced mutual encouragement and empathy. Likewise, a unilateral surveillance environment intensified peer comparison that, in turn, increased participant vigilance with mutual supervision. Similar to the few studies that examined the comparison component of social media weight management interventions [21,22,24], our results support the positive functions of peer comparison in enhancing motivations and friendly competition. However, the results also challenge a linear assumption about the positive relationship between peer comparison and weight behaviors. Group interactions underpinned by peer support, comparison, and surveillance can move toward both positive and negative directions that encourage or hinder engagement [24]. Collective tolerance for relapse, downturns in reference standards, and the tendency to withdraw after repeated failures of meeting group norms were consequences related to negative group dynamics. These results demonstrate the persuasive potential of peer influences in long-term communication, and suggest a need to attend to group norm changes in the design and moderation of social media weight management interventions.

In line with recent theoretical arguments about surveillance technology [26,30], this study identified two sources of panoptic gaze in social media. Together, public surveillance from medical professionals and participatory surveillance from peers created a reward and punishment system that reinforced self-awareness and self-monitoring in cyberspace. Those who conformed to disciplinary rules and achieved positive weight outcomes were rewarded when presenting positive figures in front of a virtual public. In contrast, punishments included displaying unsatisfactory weight outcomes and bearing the feelings of shame and guilt. Also aligned with past studies was the finding that power appeared in both surveillants’ gaze and observees’ responses to surveillance [29,47]. Although surveillance exerted a binding force on self-monitoring, participants could choose to stay in or exit from the virtual panopticon. Accordingly, there were tensions between the program’s intent to maximize user vigilance with weight control norms through panoptic surveillance and participants’ use of defensive strategies such as faking weight data and turning off notifications to reduce burden and escape from the gaze. The various types of resistance are a complex result of participants’ dissatisfaction with weight outcomes and their navigation between protecting a personal boundary and trading privacy for services. The permeation of participatory surveillance and its resonance with peer support and peer comparison may escalate online peer influences in unprecedented ways in the digital era. Nevertheless, surveillance technology also entails new issues such as the ease of data fabrication and privacy concerns that put new impediments on participant engagement. These are all worthy of further exploration and consideration.

### Practical Implications

Our findings demonstrate the importance of sustaining positive group dynamics to prolong engagement and the necessity of developing preemptive measures against resistance. Organizing more homogenous groups based on sociodemographics and health concerns (eg, pregnancy) might help group members find more commonality of interests, and deepen their interpersonal bonds that encourage support sharing, friendly competition, and mutual supervision. The level of homophily may be better controlled and tested through improved design [48]. Adding new stimuli in different phases of an intervention, and changing the duration of each phase, might help fuel participant motivations and sustain positive group dynamics. Both are worth investigating in future trials. Improving the briefing of communication time points during the opening workshop might help attenuate resistance. Finally, having face-to-face communication with physicians and peers—and witnessing actual body shape changes—would provide irreplaceable validation and aspirational motivation. Identifying innovative ways to include offline interaction components such as periodic gatherings may be considered in the future.

### Limitations

This study has three limitations. First, the qualitative nature of the study limits the generalization of the findings and the potential to test for associations among factors. Developing quantitative measures in experimental design can be the next step to verify the initial findings of the interplay of support, comparison, and surveillance revealed in this study. Second, it is unclear whether there is a difference between those who participated in the interviews and those who did not. In this study, we used maximum variation sampling to recruit men and women, young and old, and members from older and newer groups. Additional efforts were made to reach those who regained weight and those who withdrew from the WeChat groups. The diversity in the sample helped build a clear picture of social media interactions based on a wide range of experiences and observations. These actions may tackle some selection biases but cannot fully rule them out. Gaining more empirical support from future research may help validate the patterns observed in this study. Finally, social media–derived influences intervene in one’s weight control behaviors to some extent at best. Weight management as an enduring process requires the balance of an array of intrapersonal, interpersonal, and environmental factors. This study pointed out a few areas for improvement that may help enhance the positive impacts of social media influences in weight management interventions. Nevertheless, interpretations of the results should be based on a realistic view of the limitations of cybersocial influences.

### Conclusions

By inquiring into participant experiences using a weight management program for up to 2 years, this study reveals three aspects of social media influences derived from doctor-patient and peer-to-peer communication in cyberspace. Support, comparison, and surveillance play interrelated and fluctuant roles in motivating those with overweight or obesity to manage weight. When the dynamics are positive, social media communication strengthens disciplinary norms, encourages positive competition, and generates a binding force for engagement and retention. When the dynamics are negative, social media communication delays action, increases attrition, evokes resistance, and foments disengagement. From a theoretical perspective, the relationships among support, comparison, and surveillance identified in this study expand previously known aspects of social media influences, and advance understanding of the underlying processes of social media weight management interventions. From a practical perspective, this study offers empirical evidence that has implications for program improvement. Results of this study constitute a building block for the continuous advancement of employing social media technology to improve weight management and health.

## Appendix

Multimedia Appendix 1 Summary of participant demographics.
