# Supplementary material for: Interplay of Support, Comparison, and Surveillance in Social Media Weight Management Interventions: Qualitative Study
Source: JMIR Mhealth Uhealth. 2021 Mar 1;9(3):e19239. doi: 10.2196/19239 (PMC7961396; doi:10.2196/19239)
Supplement: Multimedia Appendix 1 [file mhealth_v9i3e19239_app1.doc]

**Multimedia Appendix 1.** Summary of participant demographics.

| Gender | Age Group | Weight^a^ (Kg) (BMI) | | | | % Weight Loss | Peer Moderator | Membership (Days) |
| --- | --- | --- | --- | --- | --- | --- | --- | --- |
|  |  | Pre |  | Post |  |  |  |  |
| Female | 21-30 | 112.5 | (38.9) | 88 | (30.4) | 21.8 | N | 383 |
|  |  | 101.5 | (39.2) | 46.5 | (17.9) | 54.2 | N | 716 |
|  |  | 72 | (29.6) | 64.6 | (26.5) | 10.3 | N | 109 |
|  |  | 66 | (27.8) | 70 | (29.5) | +6.1^b^ | Y | 701 |
|  | 31-40 | 86 | (33.6) | 56 | (21.9) | 34.9 | N | 702 |
|  |  | 75 | (30.8) | 73.5 | (30.2) | 2.0 | N | 376 |
|  |  | 71.5 | (27.9) | 68 | (26.6) | 4.9 | N | 383 |
|  |  | 80.5 | (32.9) | 78 | (31.8) | 3.1 | N | 708 |
|  |  | 96 | (33.2) | 90 | (31.1) | 6.3 | Y | 472 |
|  |  | 74.5 | (29.5) | 81.5 | (32.2) | +9.4 | Y | 382 |
|  |  | 68 | (25.9) | 61.5 | (23.4) | 9.6 | N | 375 |
|  |  | 75 | (28.6) | 63 | (24.0) | 16 | N | 196 |
|  | 41-50 | 125.8 | (49.1) | 103.5 | (40.4) | 17.7 | N | 708 |
|  |  | 96.5 | (37.7) | 67.5 | (26.4) | 30.1 | N | 472 |
|  |  | 85 | (33.2) | 70 | (27.3) | 17.6 | N | 557 |
|  |  | 74 | (28.4) | 67.5 | (25.9) | 8.8 | N | 694 |
|  |  | 98.4 | (41.2) | 72 | (30.2) | 26.8 | N | 700 |
|  | ≥ 51 | 97.5 | (33.0) | 68 | (23.0) | 30.3 | N | 716 |
| Male | 21-30 | 127 | (39.2) | 128 | (39.5) | +0.8 | N | 709 |
|  |  | 75 | (26.0) | 66.5 | (23.0) | 11.3 | N | 255 |
|  |  | 95 | (28.4) | 94.7 | (28.3) | 0.3 | Y | 201 |
|  |  | 73.6 | (26.1) | 65 | (23.0) | 11.7 | N | 213 |
|  |  | 98 | (33.5) | 87.5 | (29.9) | 10.7 | N | 318 |
|  | 31-40 | 95 | (31.7) | 88.8 | (29.7) | 6.5 | N | 703 |
|  |  | 146.8 | (46.9) | 150 | (47.9) | +2.2 | Y | 702 |
|  |  | 96.9 | (31.6) | 71.5 | (23.3) | 26.2 | N | 716 |
|  |  | 91.2 | (29.8) | 90 | (29.4) | 1.3 | N | 708 |
|  |  | 94 | (30.7) | 88 | (28.7) | 6.4 | Y | 213 |
|  |  | 103 | (35.6) | 99.5 | (34.4) | 3.4 | N | 549 |
|  | 41-50 | 91.6 | (30.1) | 77.8 | (25.5) | 15.1 | N | 695 |
|  |  | 104.7 | (35.0) | 101 | (33.7) | 3.5 | N | 550 |
|  |  | 83 | (27.1) | 73.8 | (24.1) | 11.1 | N | 557 |

*^a^* All weight data reported in this table were measured in the hospital. Pre-intervention bodyweight was measured after the opening workshop. Post-intervention bodyweight was measured after the interview. ^b^ The plus sign indicates weight gain.
